# A 10 year (2000–2010) systematic review of interventions to improve quality of care in hospitals

**DOI:** 10.1186/1472-6963-12-275

**Published:** 2012-08-24

**Authors:** Mary C Conry, Niamh Humphries, Karen Morgan, Yvonne McGowan, Anthony Montgomery, Kavita Vedhara, Efharis Panagopoulou, Hannah Mc Gee

**Affiliations:** 1 Division of Population Health Sciences, Royal College of Surgeons in Ireland, Dublin, Ireland; 2 University of Macedonia, Thessaloniki, Greece; 3 Institute of Work, Health and Organisations (I-WHO), University of Nottingham, Nottingham, United Kingdom; 4 Aristotle University of Thessaloniki, Thessaloniki, Greece; 5 Division of Population Health Sciences, Department of Psychology, Royal College of Surgeons in Ireland, Dublin, Ireland

**Keywords:** Quality of care, Hospitals, Interventions, Quality improvement

## Abstract

**Background:**

Against a backdrop of rising healthcare costs, variability in care provision and an increased emphasis on patient satisfaction, the need for effective interventions to improve quality of care has come to the fore. This is the first ten year (2000–2010) systematic review of interventions which sought to improve quality of care in a hospital setting. This review moves beyond a broad assessment of outcome significance levels and makes recommendations for future effective and accessible interventions.

**Methods:**

Two researchers independently screened a total of 13,195 English language articles from the databases PsychInfo, Medline, PubMed, EmBase and CinNahl. There were 120 potentially relevant full text articles examined and 20 of those articles met the inclusion criteria.

**Results:**

Included studies were heterogeneous in terms of approach and scientific rigour and varied in scope from small scale improvements for specific patient groups to large scale quality improvement programmes across multiple settings. Interventions were broadly categorised as either technical (n = 11) or interpersonal (n = 9). Technical interventions were in the main implemented by physicians and concentrated on improving care for patients with heart disease or pneumonia. Interpersonal interventions focused on patient satisfaction and tended to be implemented by nursing staff. Technical interventions had a tendency to achieve more substantial improvements in quality of care.

**Conclusions:**

The rigorous application of inclusion criteria to studies established that despite the very large volume of literature on quality of care improvements, there is a paucity of hospital interventions with a theoretically based design or implementation. The screening process established that intervention studies to date have largely failed to identify their position along the quality of care spectrum. It is suggested that this lack of theoretical grounding may partly explain the minimal transfer of health research to date into policy. It is recommended that future interventions are established within a theoretical framework and that selected quality of care outcomes are assessed using this framework. Future interventions to improve quality of care will be most effective when they use a collaborative approach, involve multidisciplinary teams, utilise available resources, involve physicians and recognise the unique requirements of each patient group.

## Background

The gap between the quality of healthcare possible and that currently provided has been referred to as a chasm [1]. The US based Institute of Medicine (IOM) has stated that healthcare should be safe, effective, patient-centred, timely, efficient and equitable. However, they also report that health systems globally have a high rate of errors and frequently fail to provide patients with quality healthcare [1,2]. Four key factors have been proposed to explain this failure: an increase in chronic conditions, poorly organised systems for healthcare delivery, limited use of information technology, and the increased complexity of care as a result of medical advances.

Variability in care provision and higher health costs have sharpened the focus on quality of care:

*'the focus on quality has intensified because of the concern that health care is costly, may sometimes be dispensed inappropriately and inequitably, and varies unduly among physicians and location'*[3]*.*

The IOM report and subsequent similar reports elsewhere have resulted in the establishment of organisations such as the Committee on the Quality of Healthcare in the US and equivalent organisations globally, with a specific remit to improve quality of care. However, despite this increased focus on quality of care, no clear academic consensus has emerged on either a definition of quality of care or the key elements of it [4-13].

Against a backdrop of rising healthcare costs, variability in care provision and an increased emphasis on patient satisfaction, the need for effective interventions to improve quality of care has come to the fore [14-16]. Our definition of quality of care is determined by a number of factors including definitions of health. The World Health Organisation has adopted a holistic view of health which incorporates aspects of mental, physical and social well-being [17]. Definitions of quality of care can be broad or narrow depending on whether our perspective is that of the patient, health professional, researcher etc. [18].

The scope of quality of care improvements depends on whether the intervention sought to improve the technical or the interpersonal aspects of care [18]. Technical care relates to the medical treatment of patients while interpersonal care refers to the communication of treatment to the patient. Interpersonal aspects of care has been highlighted as the, ‘*vehicle by which technical care is implemented’*[5] and yet interpersonal aspects of care receive less attention because of the lack of guidelines which facilitate measurement of success and an assumption that technical care is more scientific, precise and ultimately more important [5]. Also, as interpersonal care focuses on communication by health professionals, it may be the case that interpersonal interventions are met with institutional barriers such as a lack of input from health professionals.

Systematic reviews provide a method of assessing the effectiveness of strategies for health behaviour change [19]. The aim of this project is to complete a first systematic review of interventions which sought to improve quality of care in a hospital based setting. This review will collate existing evidence on interventions to improve quality of care and offer recommendations which will make future intervention studies both effective and accessible. This review has two main aims: 1) to establish what hospital based interventions have been implemented aiming to improve quality of care 2) to make recommendations to increase the accessibility and utility of future interventions

## Methods

### Search Strategy

The aim of this review was to retrieve data based articles which implemented interventions that sought to improve quality of care in adult general hospital settings between 2000 and 2010. Relevant articles were retrieved following systematic searches of the following databases: PubMed, PsychInfo, Medline, EmBase and CinNahl (see Additional file 1). Two researchers conducted the initial search by independently examining titles and abstracts. Full texts were retrieved for potentially relevant studies and these were assessed. A third researcher was consulted and reviewed texts in the case of disagreement. An independent review by a fourth researcher was undertaken on all full texts of the final included articles.

As this is the first systematic review undertaken to collate the existing evidence on interventions, the search strategy used a broad brush approach using overarching terms/keywords (Quality of Care’ and ‘Hospital’). Medical Subject Headings (MESH) terms were used in databases where appropriate. The use of overarching terms/keywords ensured that all potentially relevant articles were included in the initial screening. In all databases, the search was restricted to articles where the keywords were the major focus of the article.

### Inclusion Criteria

This search returned (n = 17,730) articles. Following duplicate removal, (n = 13,195) articles remained for screening. Included articles had to meet the following criteria:

(1) Peer reviewed data based papers in English

(2) Published between 2000 and 2010

(3) Explicitly stated that the aim of intervention must be to improve quality of care or an identified aspect of care

(4) Interventions had to have pre and post data

(5) Interventions had to be based in an adult general hospital

**Figure 1:**
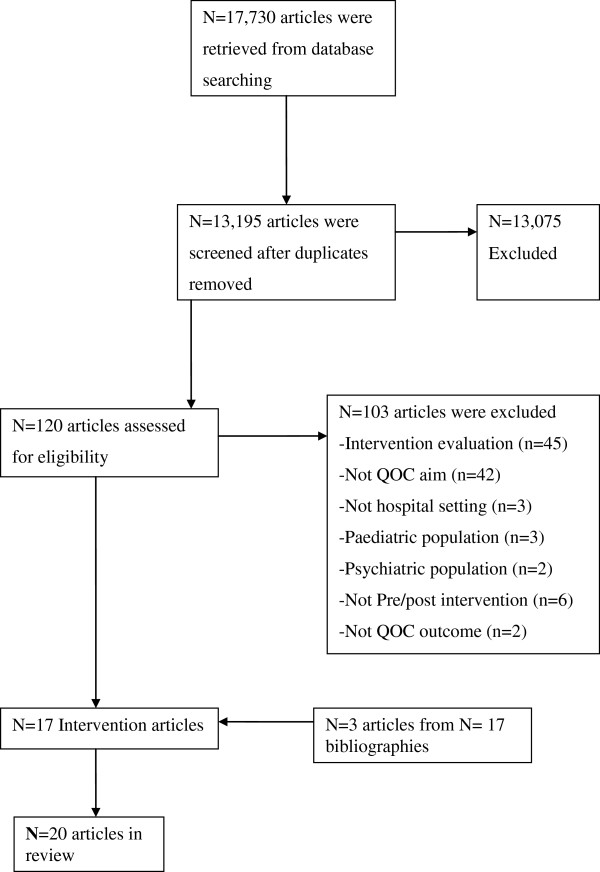
**PRISMA flow diagram of database search for data based articles on quality of care (QOC) interventions in hospital *setting*.**

To minimise bias, the above criteria were applied in a structured way to 13,195 articles. This screening process resulted in 120 articles which were examined in detail. Seventeen articles met all of the inclusion criteria and were therefore included in this review. Reference mining of the bibliographies of these articles resulted in a further 3 articles which met the inclusion criteria. The total number of articles included in the review was (n = 20). A PRISMA flow diagram summarises this screening process (Figure 1).

### Quality Assessment and Data Synthesis

The inclusion criteria permitted the inclusion of studies which were heterogeneous in terms of their design and scientific rigour. The Grades of Recommendation, Assessment, Development and Evaluation Working Group (GRADE Working Group) has developed a system for assessing methodological rigour. This approach is encouraged by BMJ and the Cochrane Collaboration [20] which have adopted the principles of GRADE for the evaluation of evidence in systematic reviews [21]. Included studies were therefore analysed using an adapted version of the GRADE criteria which assessed methodological rigour using five criteria:

1. Limitation in the design and implementation

2. Indirectness of evidence

3. Unexplained heterogeneity or inconsistency of results

4. Imprecision of results

5. High probability of publication bias

The GRADE approach specifies four levels of quality (High, Moderate, Low, Very Low). The highest quality rating is for randomized control trials and observational studies general start with a rating of low. However, if observational studies report large effects and there is no apparent bias, studies can be upgraded to moderate. Similarly, studies can be downgraded if there is evidence of bias or inconsistency.

## Results

### Included Studies

This systematic review has established that there is a very large volume of literature (n = 13,195) in the area of quality of care interventions published over the last ten years. However, the rigorous application of inclusion criteria in this study has identified a dearth of hospital based interventions at the scientific level. This systematic review identified just (n = 20) studies for inclusion in the review (see Table 1). The selected studies were heterogeneous in terms of their design and scientific rigour. The GRADE approach confirmed that there is a lack of high quality interventions (n = 1) to assess quality of care. Most of the included studies were classified as moderate quality (n = 9), low (n = 8) or very low (n = 2) (see Table 2) according to the GRADE criteria.

**Table 1:** Summary of quality of care interventions included in review

**ID**	**Study**	**Aim**	**Participants**	**Study design/Method**	**Type of intervention/Processes**	**Outcomes/ Conclusions**
A	[30] (Aghlmand et al., 2008)	•To improve the uptake of selected evidence based practices and more closely attend to identified women's needs and preferences	•n=89 women (pre-intervention) n=78 (post intervention)	Pre/post design	Interpersonal	Primary Outcome
					•Identify women’s needs, values via interviews	•Women's satisfaction levels improved significantly on 16 of 20 compared with baseline
					•Redesign care based on selected evidence-based recommendations and women's views	Other Outcomes
					•Implement the new care model	•78% of studied women experienced care consistent with the new model and fewer women had a caesarean birth
					•Measured the impact of the new care model on maternal satisfaction and caesarean birth rates utilising maternal surveys and medical record audit before and after implementation of the new care model	
						Conclusions
						•Improved compliance with evidence-based guidelines and was associated with an improvement in women's satisfaction levels and a reduction in rates of caesarean birth
B	[24] (Kalisch et al., 2007)	•To determine the impact of an intervention designed to enhance teamwork and staff engagement on the rate of patient falls, patient satisfaction, the staff’s assessment of level of teamwork on their unit, and vacancy and turnover rates	•55 staff members on the unitV 32 registered nurses (RN), 2 licensed practical nurses, 15 certified nurse assistants (CNAs), and 6 unit secretaries	•Phased design	Interpersonal	Primary Outcome
					•Focus groups were conducted to assess nature of teamwork on the unit as well as the staff educational needs in the area of teamwork	•Significantly lower patient fall rate staff ratings of improved teamwork on the unit
					•Focus group data were compiled into a report which was presented in several feedback	
					•Each staff member then attended a day-long team training program	Other Outcomes
						•Lower staff turnover and vacancy rates .
						
						•Patient satisfaction ratings approached, but did not reach, statistical significance
						
					•Rapid testing of ideas	
						Conclusions
						•There is a continual need to work with staff in the areas of listening, feedback and conflict management
C	[27] (Curtis et al., 2008)	•To improve palliative care in the ICU	•Patients who died in the ICU were identified pre- (n= 253) and post-intervention (n=337)	Pre/post design	Interpersonal	Primary Outcome
						•The family-QODD, showed a trend toward improvement but was not statistically significant Family satisfaction increased but not significantly
					•The intervention consisted of clinician education, local champions, academic detailing, feedback to clinicians, and system support	
						Other Outcomes
						•The nurse-QODD showed significant improvement and there was a significant reduction in ICU days prior to death (pre 7.2, post 5.8; p<0.01)
					•Families completed Family Satisfaction (FS-ICU) and Quality of Dying and Death (QODD) surveys.	
						Conclusions
						•Improving family ratings may require interventions that have more direct contact with family members
D	[26] (Kipp et al., 2001)	•To improve patient satisfaction, a significant quality outcome measure for healthcare providers	•500 bed community hospital	Pre/post design	Interpersonal•A multidisciplinary group was formed and comprised ED physicians, RNs, technicians, clerical staff, managers, and human resource development personnel •The group met monthly from April 1998 to October 1998 to develop the Nursing Caring Standards•The standards were derived from four previously established Department of Nursing Caring Standard	Primary Outcome
						•ED patient satisfaction with the "care and concern by nurses" increased 6.6% after the caring standards were implemented
						Other Outcomes N/A
						Conclusions
						•The development of concrete ED customer service standards appears to be effective in improving caring behaviours by staff and patient satisfaction
E	[25] (Oosthuizen et al., 2002)	•To improve the quality of care for diabetic patients	•n=23 doctors Phase 1 (n=31 patients) Phase 2 (n=32 patients)	Pre/post design	Interpersonal•A Diabetes Attitude Scale (DAS-3) and a Diabetes Practice Scale (DPS) were completed by each doctor before and after the interventional educational sessions •Data from diabetic patients in the wards were collected for 5 weeks before and 5 weeks after the interventional training •These two sets of data were compared to measure the effect of the interventional training	Primary Outcome
						•Subscales of the DA5-3 showed a statistically significant improvement in attitude regarding seriousness of diabetes mellitus
						Other Outcomes
						•A trend towards improvement in attitude regarding need for special
						training and patient autonomy
						•Most of the items on the DPS improved significantly
						Conclusions
						•A short educational intervention resulted in an improvement in attitude, knowledge and clinical management of diabetic patients
F	[29] (Brown et al., 2007)	•To encourage uptake of childbirth companions in state hospitals	•Maternity staff at n=10 hospitals •n=200 women	RCT	Interpersonal •Educational intervention to promote childbirth companions	Primary Outcome
						•No effect was demonstrated on the number of women having a companion
						Other Outcomes
						•No effect on being shouted at, left alone, not offered food or fluids or physically mistreated
						•There was a statistically significant reduction in episiotomy
						•Fewer women reported being mobile during the second stage of labour at the intervention hospitals
						Conclusions
						•Unable to determine whether the presence of a lay carer impacted on the humanity of care provided by health professionals
G	[28] (Schmied et al., 2009)	•To design, implement and evaluate strategies to improve the quality and content of hospital-based postnatal care	•146 women at baseline and 148 women post intervention completed a postal self-report questionnaire between 2–4 weeks postpartum	Pre/post design	Interpersonal •Compared the effect of multifaceted strategies on perceptions of quality and content of postnatal care, knowledge and experience of postnatal problems, parenting self-efficacy and breastfeeding outcomes •Key strategy implemented, ‘One-to-one time’, focused on providing women an uninterrupted period of time each day when a midwife would be available to discuss women’s concerns	Primary Outcome
						•No significant differences between baseline and post intervention groups in perceived quality of care, breastfeeding outcomes and maternal self-efficacy
						Other Outcomes
						•Women experiencing health issues were more likely to report that they received good or excellent care post intervention
						•Women were less likely to report excessive tiredness postintervention
						•‘One-to-one time’ was not consistently implemented.
						Conclusions
						•Is potential for individualised care but institutions are difficult to change
H	[23] (Moffitt et al., 2009)	•To increase patient, physician, and staff satisfaction and to improve patient outcomes	•Not stated	Phased design	Interpersonal • Merger of a medical-oncology unit at a small community hospital	Primary Outcome
						•The Medical unit demonstrated improvement in overall patient satisfaction
						Other Outcomes
						•A decrease in the change of shift report time and a staff that desires empowerment
						Conclusions
						•The results of the changes implemented on an medical oncology unit indicated improvements in physician, patient and nurse satisfaction
I	[22] (Wessels et al., 2010)	•To address the effect of an intervention in hospital structure (integration of three units into one) with the purpose of improving processes (increase meeting, cooperation and communication between professionals and patients) and its effect on the outcome (cancer patient satisfaction)	•Cancer patients (n=174, n = 97	Pre/post design	Interpersonal •Physical integration by bringing separately located units (outpatient clinic, day-care clinic, clinical ward) together in one wing of the hospital and adjustments in communication and coordination structures	Primary Outcome
						•Patient satisfaction with care improved for six scales
						Other Outcomes
						•The most important improvement was found at the day-care clinic on aspects like ‘the degree in which the nurses were informed about a patients situation’, ‘privacy’, ‘interior design’, ‘quality of hospital equipment’,‘sanitary supplies’ and ‘waiting periods’.
						•With regard to continuity and coordination of care, satisfaction increased for five items
						Conclusions
						Integration of three oncology units into one unit had a positive impact on care delivery processes and resulted in improved patient satisfaction concerning care and treatment
J	[37] (Varelas et al., 2004)	•To evaluate the impact of a newly appointed neurointensivist	•n=1,087 patients before appointment of neurointensivist and n=1,279 after	Observational cohort with historical controls	Technical •Analyzed patients before and after the neurointensivist’s appointment	Primary Outcome
						•Unadjusted in-hospital mortality decreased
						Other Outcomes
						•Discharge home increased
						•Significant reduction in risk of death during first 3 days of admission
						Conclusions
						•The institution of a neurointensivist-led team model was associated with an independent positive impact on patient outcomes
K	[35] (Nolan et al., 2005)	•To improve the quality of care for patients with acute myocardial infarction and heart failure	•n=Not stated	Phased design	Technical •Multidisciplinary initiative with a partnership of inpatient cardiology nursing and physician leadership	Primary Outcome
						•Dramatic trend upward in the discharge teaching and smoking-cessation counseling, Other Outcomes
					•This inpatient leadership team analyzed clinical and operational processes, and revised and developed tools such as standard order sets, discharge instructions, clinical pocket guides, and daily monitoring logs	
						•Improvement in angiotensin-converting enzyme inhibitor use and left ventricular ejection fraction measurement
						Conclusions
						•At 12 months, quality improvements have been demonstrated
L	[34] (Scott et al., 2000)	•To improve quality of in-hospital care of patients with acute coronary syndromes	•n=1,594 from 3 hospitals	•Pre/post design	Technical	Primary Outcomes
						•Increases occurred in the proportions of eligible patients: (i) undergoing timely ECG (ii) prescribed angiotensin-converting enzyme inhibitors and lipid-lowering agents
					•Multi-improvement program: Clinical guidelines, reminder tools, and educational interventions; 6-monthly performance feedback; pharmacist mediated patient education program; and facilitation of multidisciplinary review of work practices	
						Other Outcomes
						(iii) Increase in the number receiving cardiac counselling in hospital and referred to cardiac rehabilitation
						Conclusions
						•Multifaceted approaches can improve care processes for patients hospitalized with acute coronary syndromes.
						•Care processes under direct clinician control changed more quickly than those reliant on complex system factors
M	[39] (Van Zyl et al., 2004)	•To determine if a physician education programme and a structured consultation schedule would improve the quality of diabetes patient care in a diabetes clinic	•n=141 patient and n=159 control	•Pre/post design	Technical	Primary Outcomes
					•Three hundred patients were randomly selected for audit of their hospital records: 141 from the intervention and 159 from the control clinics	
					•Thereafter a physician training programme and a structured consultation schedule were introduced to the intervention clinic and maintained for a 1-year period	
					•The control clinic continued with care as usual. Process and outcome measures were determined at a post-intervention audit and compared between the two groups	
						•After intervention the intervention group had significantly higher process measure scores than the control group. HbA1c did not significantly differ between the two groups
						Other Outcomes
					•Consultation time was measured for both the intervention and control groups and data were compared	
						•The average number of clinic visits reduced over time for the intervention group compared with the control group, but the average consultation times were significantly longer
						Conclusions
						•The introduction of a physician education programme and a structured consultation schedule improved the quality of care delivered at a tertiary care diabetes clinic
N	[36] (Feldman et al., 2006)	•To improve the quality and consistency of care by adapting and adopting national guidelines	•1 academic medical college (November 2002 –July 2003)	Phased design	Technical	Primary Outcomes
					•Multidisciplinary program	
					•Initiation phase, diagnostic engagement phase, design phase, implementation phase	
						•Improvement in several quality measures including increased use of beta blockers and angiotensin converting enzyme inhibitors for heart failure patients
						Other Outcomes
						•Reduced length of stay for heart failure and acute coronary syndrome patients, and increased satisfaction of the clinicians
						Conclusions
						•Individual physician’s unwillingness to embrace change was overcome with the development of faculty leadership skills and enhanced physician accountability
O	[33] (Mehta et al., 2002)	•To measure the effects of a quality improvement project on adherence to evidence-based therapies for patients with AMI	•Medicare and non-Medicare patients at baseline (n=735) and (n=914) at remeasurement	Pre/post design	Technical	Primary Outcomes
					•The GAP project consisted of a kickoff presentation; creation of customized, guideline-oriented tools designed to facilitate adherence to key quality indicators	
					•Identification and assignment of local physician and nurse opinion leaders; grand rounds site visits	
					•Premeasurement and postmeasurement of quality indicators	
						•Increases in adherence to key treatments were seen in the administration of aspirin and blockers on admission and use of aspirin and smoking cessation (counseling) at discharge
						Other Outcomes
						•For most of the other indicators, nonsignificant but favorable trendstoward improvement in adherence to treatment goals were observed.
						• Medicare patients in GAP hospitals showed a significant increase in the use of aspirin at discharge
						• Use of aspirin on admission, ACE inhibitors at discharge, and documentation of smoking cessation also showed a trend for greater improvement among GAP hospitals compared with control hospitals, although none of these were statistically significant
						Conclusions
						•Implementation of guideline-based tools for AMI may facilitate quality improvement among a variety of institutions, patients, and caregivers
P	[31] (Halm et al., 2004)	•To evaluate the impact of a multifactorial intervention to improve the quality,efficiency, and patient understanding of care for community-acquired pneumonia	•Four academic health centres (n= 1,013) before intervention and (n=1,081) after	•Time series cohort	Technical	Primary Outcome
						•Increased the use of guideline recommended antimicrobial therapy
						Other Outcomes
						•Borderline decrease in the proportion of patients being discharged prior to becoming clinically stable
					•A multidisciplinary team of opinion leaders developed evidence-based treatment guidelines and critical pathways, conducted educational sessions with physicians, distributed pocket reminder cards, promoted standardized orders, and developed bilingual patient education materials	
						•No improvements in the other targeted indicators, including time to first dose of antibiotics, proportion receiving antibiotics within 8 h, timely switch to oral antibiotics, timely discharge, length of stay, or patient education outcomes
						Conclusions
						•Modest improvement on some quality indicators, but no effect on resource use or patient knowledge of their disease
Q	[32] (Meehan et al., 2001)	•To improve process-of-care performance and to decrease length of stay for patients hospitalized with community-acquired pneumonia	•n=1,242 patients at baseline, n=1,146 at follow up	•Pre/post design	Technical	Primary Outcomes
					•Interventions included feedback of performance data, dissemination of an evidence-based pneumonia critical pathway and sharing of pathway implementation experiences (hospitals)	
						•Improvements were noted in antibiotic administration within 8 hours of hospital arrival, oxygenation assessment within 24 hours of hospital arrival and length of stay 7 days to 5 days
						Other Outcomes
						•There were no significant changes in blood culture collection within 24 hours of hospital arrival, blood culture collection before antibiotic administration, 30- day mortality, or 30-day readmission rates
						Conclusions
						•Statewide improvements were demonstrated in the care of hospitalized pneumonia patients concurrent with a multifaceted quality improvement intervention
R	[38] (Choma et al., 2009)	• To improve hypertension care at Veterans Affairs– Tennessee Valley Healthcare System	•2 teaching hospitals, 5 community-based outpatient clinics, and 4 contract clinic sites	Pre/post design	Technical	Primary Outcome
					•Multiple Interventions	
						•There was an absolute improvement of 4.2% in BP
						Conclusions
					•Observation time was 40 weeks (14 weeks preintervention, 8 weeks intervention implementation, and 18weekspostintervention), during which there were 55 586 unique clinic visits for hypertension	
						•After implementing small, focused, and inexpensive interventions, BP control improved 4.2%, thereby improving the quality of hypertension care
S	[41] (Koplan et al., 2008)	•To assess the effect of adding tobacco order set to an existing computerized order-entry system	•7,278 of 17,530 admissions	Pre/post design	Technical	Outcomes
						•Intervention increased the proportion of admitted patients who were referred for smoking counselling and had Nicotine Replacement Therapy ordered
					•Adding a brief tobacco order set to an existing computerized order-entry system	
						Other Outcomes
						•Hospital’s performance on the smoking cessation quality measure improved
						Conclusions
						•Hospital’s provision of evidence-based tobacco treatment helped to improve its performance on a publicly reported quality measure
						•Provides a model for US hospitals seeking to improve their quality of care for inpatients
T	[40] (Smith et al., 2004)	•To use a focused change programme (the Better Births Initiative) to influence obstetric practice at 10 hospitals in Gauteng, South Africa	•Postnatal women were at baseline (n = 247) and •Follow-up (n = 215) focus group discussions (n= 8) with labour ward staff •Key labour ward staff at each site (n = 14).	Pre/post design	Technical	Primary Outcomes
						•Providers at some sites reduced the use of enemas, shaving and episiotomy
						Other Outcomes
					•Workshops for staff on obstetric practices	
						• Increased use of oral fluids and companionship during labour
						Conclusions
						•An interactive approach to implementing evidence-based practice can influence health professionals' decisions to change practice, and that good working relationships and enthusiastic staff are central to effective change

**Table 2:** GRADE assessment of included studies

**ID**	**Study**	**Limitations of design (Risk of bias)**	**Inconsistency or heterogenity**	**Indirectness (PICO and Applicablity)**	**Imprecision of result**	**Publication bias**	**Quality rating**
A	[30] (Aghlmand et al., 2008)	√	√	√	√	√	Moderate
B	[24] (Kalisch et al., 2007)	√	√	√	X	√	Low
C	[27] (Curtis et al., 2008)	√	√	√	√	√	Moderate
D	[26] (Kipp et al., 2001)	√	X	X	X	√	Very Low
E	[25] (Oosthuizen et al., 2002)	√	√	√	X	√	Low
F	[29] (Brown et al., 2007)	√	√	√	√	√	High
G	[28] (Schmied et al., 2009)	√	√	√	X	√	Low
H	[23] (Moffitt et al., 2009)	√	√	√	X	√	Low
I	[22] (Wessels et al., 2010)	√	√	√	X	√	Low
J	[37] (Varelas et al., 2004)	√	√	√	√	√	Low
K	[35] (Nolan et al., 2005)	√	√	X	√	√	Low
L	[34] (Scott et al., 2000)	√	√	X	√	√	Moderate
M	[39] (Van Zyl et al., 2004)	√	X	√	√	√	Low
N	[36] (Feldman et al., 2006)	√	X	√	√	√	Very Low
O	[33] (Mehta et al., 2002)	√	√	√	√	√	Moderate
P	[31] (Halm et al., 2004)	√	√	√	√	√	Moderate
Q	[32] (Meehan et al., 2001)	√	√	√	√	√	Moderate
R	[38] (Choma et al., 2009)	√	√	√	√	√	Moderate
S	[41] (Koplan et al., 2008)	√	√	√	√	√	Moderate
T	[40] (Smith et al., 2004)	√	√	√	√	√	Moderate

### Study Characteristics

Details of all studies (n = 20) included in the review and a summary of the data abstracted are displayed in the data was extracted using the PICO approach. The majority of studies were described as pre/post design (n = 13) and the remaining studies used a phased design (n = 4), observational design (n = 1), time series cohort (n = 1) or randomised controlled trial (n = 1). Included interventions review varied in scale from small scale improvements for specific patient groups in individual settings to large scale quality improvement programmes across multiple settings. As discussed earlier, studies were grouped into two categories: Interpersonal and Technical.

#### Interpersonal Quality of Care Interventions (n = 9)

Nine studies (see Table 2 A-I) focused on improving the interpersonal aspects of care for specific patient groups such as cancer patients [22-24], diabetic patients [25], patients in the emergency department [26], palliative care patients [27] postnatal care patients [28] and women during childbirth [29,30]. Patient satisfaction with care was prioritised in those articles which sought to intervene in the delivery of interpersonal care [22,23,25-27,30] while two studies sought to improve both patient and staff satisfaction [23,24].

### Implementation

Implementation of interventions was assessed by establishing who carried out the intervention. While the participant groups involved in these interventions varied, interpersonal interventions were predominantly implemented or carried out by nursing staff. Four interventions were led exclusively by nursing staff [23,24,26,28] while one intervention was implemented by midwives and physicians [30]. Quality of care outcomes from interventions implemented by nurses were measured using patient satisfaction questionnaires [23,24,26,28,30].

Clinicians implemented the interventions in two studies [25,27]. In these studies, quality of care outcomes were measured using nurse and family satisfaction questionnaires [27] and a Diabetes Attitude Scale (DAS-3) [25]. In contrast to the majority of interpersonal interventions, two studies differed in terms of participants with one study involving patients and childbirth companions [29] while the second study assessed the impact of building restructuring on quality of care [22]. Despite having the differences, both studies [22][29] measured quality outcomes used patient satisfaction questionnaires.

### Intervention Structure

Interpersonal interventions were described as having either a multifaceted structure involving several components or having one central component i.e. an educational intervention. However, two intervention studies [22,23] were unique in that they sought to improve quality of care by altering the physical structure of the hospitals. Both studies sought to improve quality of care by integrating separate oncology clinics into one unit within the hospital.

Three studies implemented interventions which were multifaceted in design [27,28,30]. These interventions sought to improve various aspects of maternity care [30], postnatal care [28] and palliative care [27]. Similar approaches were adopted by the interventions which sought to improve maternity care [28] and postnatal care [30]. In both studies, baseline data was collected to identify patient needs and a medical team then redesigned care processes based on those needs.

The study examining postnatal care encouraged parental self-efficacy by providing ‘one to one’ time with the midwife each day so that the woman could discuss her concerns and gain knowledge [28]. The study [30] which sought to improve childbirth implemented a care model based on five criteria (availability of resources, the physical environment of the maternity ward, clinical experience and culture and correspondence with women’s needs and requirements). Similarly, a multifaceted interdisciplinary intervention to improve palliative care identified five key components of an effective intervention-clinician education, local champions, academic detailing, feedback to clinicians and system support [27]. This intervention was based on the theory of self efficacy and it was hypothesised that changes in attitudes, behaviour and knowledge of clinicians would improve palliative care.

Educational workshops or training for staff were a central component in four of the interpersonal intervention studies [24-26,29]. The aim of the training/education in all cases was to increase knowledge so that the delivery of care and care processes could be improved. Staff who received training/education included maternity staff [29], emergency department staff [26], nursing staff [24] and doctors [25].

### Intervention Outcomes

Reported quality of care outcomes or improvements were varied across the nine interpersonal studies [22-30]. An educational intervention targeted at doctors to improve diabetes care reported improvements in the knowledge, attitude and clinical management of diabetic patients [25] while the improvement in patient satisfaction was statistically significant in only one of the educational interventions [26]. In contrast, patient satisfaction approached but did not reach statistical significant in an intervention which sought to improve teamwork and staff engagement although, reduced staff turnover, improved teamwork and lower patient fall rates were reported. [24].

Multifaceted interventions reported improvements in care for women during childbirth [30]. In a study which aimed to improve palliative care, nurse satisfaction improved but family satisfaction did not reach statistical significance [27]. A multifaceted approach to improve postnatal care reported no significant differences post intervention in perceived quality of care [28] however it was reported that, the key strategy of ‘one to one time’ for patients had not been implemented consistently. Similarly, an educational intervention to encourage the presence of childbirth companions found no significant difference in patient satisfaction or humanity of care based on whether a companion was allowed by nursing staff [29]. In summary, interpersonal interventions tended to be focused on patient satisfaction and were implemented by nursing staff. They also tended to be multifaceted or involve education/training. Most reported some improvements in patient satisfaction but not all findings reached statistical significance.

#### Technical Quality of Care Interventions (n = 11)

Ten studies (see Table 1 J-T) sought to improve technical aspects of care. Technical interventions focused on improving medical outcomes for patients with pneumonia [31,32] or myocardial infarction related illnesses [33-36]. Technical interventions were also implemented to improve care for specific patient groups including those in intensive care [37], patients with hypertension [38], patients with diabetes [39] and postnatal women [40]. One intervention sought to promote smoking cessation in patients who were identified as smokers at admission [41].

### Participants

Five technical interventions set up a team or panel of experts prior to the intervention [31-34,38]. Teams tended to be multidisciplinary and had the task of setting goals and reaching consensus on quality indicators prior to intervention. Five technical interventions were implemented by physicians [36,37,39-41], while in one study [35] the intervention was implemented by both nurses and physicians.

### Intervention Structure

Technical interventions tended to involve a number of interconnecting components [31-34,38]. A multifaceted intervention [31] sought to improve pneumonia care took place in multiple centres although the data collection was predominantly hospital based. Three studies implemented quality improvement programs which aimed to improve hypertension care [38] and care of patients with heart diseases [34,35]. Similarly, two studies implemented multifaceted interventions but these interventions were part of state-wide initiatives including the ‘Pneumonia Pathway Project’ [32] and the ‘Guidelines Applied in Practice’ GAP initiative to improve care of patients with myocardial infarction [33]. Four of the technical interventions had structural similarities in that they were all implemented by physicians and sought to alter care processes [36,37,39,41]. One intervention altered care processes for diabetes patients by implementing a diabetes education workshop for doctors [39]. Another [40] intervention sought to improve evidence based practice for women during labour by implementing workshops for obstetric practices for staff. Interventions implemented by physicians included the addition of a tobacco order set to an existing computerized order entry [41], the appointment of a new neurointensivist team to an intensive care unit [37] and the adoption of myocardial infarction guidelines [36].

### Intervention Outcomes

Multifaceted interventions reported improvements in quality of care with an absolute improvement in blood pressure control in a study to improve hypertension [38]. Three multifaceted interventions [33-35] aimed to improve quality of care for patients with heart disease and reported improved medical outcomes including hospital administration of key treatments such as aspirin at admission [33] and improvement in angiotensin [35]. An intervention [34] to improve acute coronary care reported improvement of key quality indicators including timeliness of treatment but found no significant change in the proportion of patients accessing treatments such as antiplatlet agents or undergoing coronary angiography.

Technical interventions [31,32] which sought to improve pneumonia care reported some quality of care improvements with an increase in the use of guideline recommended antimicrobial therapy [31] and antibiotic administration within eight hours [32]. However, it was reported that there was no significant improvement in indicators such as timeliness and patient education in one study [31] and no significant improvement in indicators such as thirty day mortality and thirty day readmission in the other study [32].

Interventions implemented solely by physicians reported quality of care improvements. An increased number of patients accessed NRT or smoking counsellors after a computerised order entry form introduced for use by doctors [41]. Care improved for diabetes patients as a result of a physician education programme [39] and medical outcomes for women during childbirth improved as a result of an educational programme on obstetric practices for staff [40]. Mortality outcomes for patients in intensive care improved following the appointment of a neurointensivist [37] and quality measures for heart diseases improved after a multidisciplinary programme was implemented [36]. In summary, technical interventions were mainly implemented by physicians and concentrated on improving care for patients with specific conditions such as heart disease or pneumonia. Multidisciplinary panels of experts were formed to set goals and reach consensus on quality indicators prior to intervention. Technical interventions tended to achieve improved medical outcomes for patients with specific illnesses.

## Discussion

Significant strides have been made in health research particularly in the area of hospital based quality improvement. The strength of this review is that it is the first systematic attempt to collate and appraise the very large volume of literature on quality of care interventions over a ten year period. This review has established that despite the volume of literature, there is a paucity of hospital interventions with a theoretically based design or implementation.

The broad scope of the review search strategy resulted in the inclusion of a diverse range of interventions in terms of scope and scientific rigour. Studies varied from small scale improvements for specific patient groups to large scale quality improvement programmes across multiple settings. This heterogeneous group of interventions is a product of the rigorous adherence by the researchers to the review inclusion criteria. This approach succeeded in highlighting a number of areas for improvement for future quality of care interventions.

The inclusion of heterogeneous interventions in this review meant that data synthesis was limited to broad qualitative descriptions of the main components of interventions. Interventions were broadly categorised into two categories. Interpersonal interventions sought to improve patient satisfaction and tended to be implemented by nursing staff while technical interventions were generally implemented by physicians and reported measurable improvements in medical outcomes for patients with specific illnesses. There was a tendency for both categories of interventions to focus on evaluating outcomes without due regard to the mechanisms that produced these outcomes. The result was that interventions appeared to select quality of care outcomes on an ad-hoc or local basis and this arbitrary selection of outcomes makes measurement and comparison of quality of care outcomes difficult.

Technical interventions had a tendency to achieve more substantial improvements in quality of care. This may be because improving and measuring improvements in technical aspects of care is more straightforward and precise than interpersonal aspects of care. When physicians implement interventions to improve processes of care, they tend to have independent control over those processes and this makes implementation of change easier [31]. Also, it is suggested in the literature that physicians are more likely than other health professionals to alter their behaviour when the outcome will affect the medical outcomes of their patients such as mortality [31,32] or perhaps physicians were more likely to identify outcomes which they felt confident that they could actually improve.

Difficulties in achieving quality improvements may also be related to external factors such as administration with one of the major challenges in implementing an intervention to improve teamwork cited as the lack of administrative support [23]. However, they stressed that when staff are empowered, quality improvements were made. One study concluded that organisational support for change should be achieving by offering financial incentives in the form of salary increments [36].

One of the acknowledged shortcomings in interpersonal interventions to improve maternity care was the failure to appreciate the difficulties in achieving organisational change [29,30]. The authors concluded that maternity care interventions would be more successful when they adopted multifaceted approaches which involved various stakeholders [29,30]. In contrast, one of the main strengths of technical interventions was the involvement of teams or panels of experts prior to intervention [31-34,38]. This approach helped to identify local barriers, establish key areas for quality improvement and establish a plan for achieving manageable tasks [38]. The use of expert panels acted as an integral part of state wide interventions as this approach facilitated the alignment of resources and expertise from multidisciplinary organisations [32].

Interpersonal interventions stressed the importance of recognising the views of the patient prior to intervention [27,29,30]. In maternity care, it was established that this patient group are aware of their own needs and that this information will be valuable in designing future quality improvement programmes [30]. Similarly, if nursing staff are implementing an intervention to encourage the uptake of childbirth companions, they should be interviewed prior to intervention to provide an insight into the potential barriers to the intervention [29]. Also, if an intervention seems to improve care for two groups such as patients and family members, it is imperative that the different needs of both groups are recognised. An intervention to improve both staff and family satisfaction acknowledged that while it achieved improved staff satisfaction, it failed to achieve improved family satisfaction as the intervention lack components which directly targeted family members [27].

In response to the need for effective interventions, the Medical Research Council UK Framework has released guidelines stating that interventions need a clear theoretical basis to inform their hypothesis. This increased emphasis on the importance of a theoretical base for interventions will facilitate the development and evaluation of interventions [42]. The majority of studies excluded from this review neglected to mention the theoretical basis of the intervention or to identify their position along the quality of care paradigm. This is the primary reason why the number of studies included in this review was small relative to the very large volume of literature. In light of this lack of clarity, it is suggested that the lack of theoretical grounding of intervention studies may partly explain the minimal transfer of health research into health policy [43].

The findings of this review and those of other authors suggest that collaborative research is a key strategy for implementing future theory based interventions [43]. Collaborative research encapsulates the expertise of all relevant stakeholders (academic researchers, hospital management, patients and their families and policy makers). In this way the theoretical basis of the intervention is not solely based on the perspectives of those who are implementing the intervention. The contributions of policy makers and hospital management ensure that interventions which reach implementation stage are those which are most cost-effective and sustainable in the long term.

### Limitations

The results of this review must be interpreted with caution. As this was the first systematic review of its kind, a broad reaching search strategy was necessary in order to capture all potentially relevant studies. One of the disadvantages of this search strategy was that studies of heterogeneous design were included which resulted in the use of a modified version of the GRADE criteria for quality assessment.

The inclusion of studies of varied design and scientific restricted us to presenting a broad assessment or overview of studies. Different approaches were explored for presenting the studies in a meaningful way. While interventions in the main focused on improving either technical or interpersonal aspects of care, there was overlap with some interventions seeking to improve patient satisfaction along with medical outcomes. However, interventions categorised as technical reported primary medical outcomes and interpersonal interventions reported interpersonal primary outcomes.

The majority of interventions included in this review were pre/post design. Results of any before and after study must be interpreted with caution. In hospital based pre-post interventions, it is often the case that participants at time one and time two differ and this can have the effect of diluting the intervention effects. Improved outcomes reported in the pre/post designed intervention studies may have several possible explanations including secular or temporal trends. The most effective method of overcoming this possibility is to use a randomized controlled trial (RCT). However, using RCTs is difficult when implementing complex interventions involving multiple components since it is not possible to ‘blind’ providers or recipients to the control and intervention groups and it is also difficult to establish which components of a complex intervention worked and which did not.

## Conclusions

This review has established that despite the very large volume of literature, there is a paucity of hospital based interventions with a theoretically based design or implementation. Intervention studies to date have largely failed to identify their position along the quality of care spectrum and it is suggested that this lack of theoretical grounding may partly explain the minimal transfer of health research into health policy. It is necessary to ground future interventions within an established theoretical framework and to assess selected quality of care outcomes using this framework. This review concludes that a collaborative approach is necessary in future interventions to increase the utility and effectiveness of interventions to improve quality of care. Future interventions to improve quality of care will be most effective when they adopt this collaborative approach, use multidisciplinary teams, utilise available resources, involve physicians and recognise the unique requirements of each patient group.

## Competing Interest

The authors declare that they have no competing interests.

## Author Contributions

MC- Acquisition of data, analysis and interpretation of data, drafting of manuscript, NH- Acquisition of data, analysis and interpretation of data, critical revision of paper, YM- Acquisition of data, analysis and interpretation of data, critical revision of paper, KM- Conception and design, analysis and interpretation of data, critical revision of paper, KV- Analysis and interpretation of data, critical revision of paper, AM- Conception and design, analysis and interpretation of data, critical revision of paper, EP- Conception and design, analysis and interpretation of data, critical revision of paper, HM- Conception and design, analysis and interpretation of data, critical revision of paper. All authors read and approved the final manuscript.

## Pre-publication history

The pre-publication history for this paper can be accessed here:

http://www.biomedcentral.com/1472-6963/12/275/prepub

## Supplementary Material

Additional file 1 Search Strategy results. Click here for file
